# Annotations

**Published:** 1897-08-14

**Authors:** 


### Annotations.

### Seaside Dangers.

All the world is now flocking out of town in search of renewed health and strength to be gained on the coast and in the country. Just before packing up one's hopes in one's portmanteau it may be worth while to ask if they are well founded. We give up a commodious, comfortable house, the sanitation of which is as perfect as science can make it, and go to some lodging which may indeed be clean and neat so far as the eye can judge, but concerning the drains of which nothing can be known. It is as easy to inhale sewer-gas as ozone, and that this is done is the reason why people so often come home rather the worse than the better of their holiday. In a case like this the visitor can hardly be blamed for neglecting the obvious duty of self-protection; but disease instead of health is often found in circumstances in which care might, apparently, be more easily exercised. How many people ask their prospective landlady who her last tenants were? If they do make any such inquiry, are they not satisfied with some assurance as to the social status and respectability of their predecessors, instead of demanding, as they should, a health chart. It might be that the landlady would not know that the previous occupant of her rooms was recovering from diphtheria or scarlet fever, or if she knew, would answer the question falsely. But surely it would not be impossible to make a law compelling an intending lodger to state if, within a specified time preceding his occupancy, any of his family had suffered from infectious disease. Complaisant landladies might not be too anxious to demand this certificate at first, but they would learn to do so if they had to pay damages to anyone who caught an illness under their roof which could be traced to their negligence. The friends of convalescents might tell lies, but they would cease to do so if they in turn were liable to a penalty for false statement. At first, perhaps, there would be an outcry, but the regulations once laid down would finally be accepted as a matter of course; and the only change would be that convalescents would not leave home until they had a medical certificate that others were not liable to be infected through contact with them or anything they had used.

### A Good Word for Tricycles.

The bicycle girl is as conspicuous as ever, and even though the fashionable world may give up its craze for the flying wheel, it is safe to say that cycling will remain with us not merely as a pastime but also as a means of locomotion. So much the better; but, unfortunately, bicycling benefits only those of the female sex who are sure to take exercise enough already—who would play tennis or golf, or walk, or row, if wheeling had never been invented. Far more necessary is some form of exercise for the matron of forty or more, who after her household work is done inclines to sit down with a book or a piece of needlework, and drop into a doze from sheer *ennui*. Her doctor tells her to take exercise, but she gets tired of walking the mile or two round her own door, which is the utmost she can manage; and a carriage, even if she possesses one, may give her air but hardly exercise. Some even of these ladies have learned the bicycle, but there are others who fear the effort of learning, and doubt if they could ever balance the wheel. Why then do they not take to the tricycle? The tricycle is less graceful than the "bike," it is less swift, and it does not give the fascination of performing a seemingly impossible feat; but it has its merits. It requires no learning; it can be arranged to carry any reasonable quantity of luggage, which makes it an ideal vehicle for country shoppers, who are sure to have endless little errands when they go to their market town; and, not least, if you get tired you have only to sit still until you recover breath, instead of coming off and having to support your helpless steed. Altogether, a tricycle is a very convenient machine.

### Grocers and Adulteration.

As a trade organ the *Grocer* is an admirable journal, and sticks up manfully for the interests of its constituents. In so doing it has fallen foul of The Hospital, and has poured out its wrath upon us for even supposing that grocers adulterate their goods. The matter arose in this wise: A little time ago we had occasion to draw attention to what we ventured to call the ridiculous arrangement under which samples of food are obtained by the authorities for purposes of analysis. Incidentally we, of course, assumed that such a thing as adulteration did exist, and herein lies our great offence. We fancy, however, that in this matter we are in agreement with the majority of those who have thought about the matter; certainly we have on our side the Legislature, which has gone to the trouble of passing special laws to restrain this peculiar form of cheating. Perhaps this is a trifle; perhaps we ought to accept the assurance of the *Grocer* as to the innocence of the trade, and to look upon laws and inspectors and prosecutions as the outcome of mere prejudice. We, however, are great respecters of the law, and while all this machinery for the suppression of adulteration is maintained at the expense of a tax-paying public we must fain believe in the existence of the evil it is intended to cope with. So much for criticisms of the *Grocer*. When, however, we turn to what was the main point of our article, viz., that the present system of obtaining samples for analysis was not only ridiculous but was in some instances "lowering to the dignity of the law," we are glad to find that the *Grocer*, which, however prejudiced it may be as to the virtues of its constituents, is reliable enough as to the details of the trade, writes as follows in regard to the system we denounced. "An inspector begins his work with the specific idea of getting up a 'case.' That is the only object he has in view. Whether he is known or unknown to the shopkeeper is a point of small importance, for he does not make the purchases himself. He prefers to employ shabby agents, perhaps an old man out of work, or a young girl of undeveloped intellect. He tells them what to do, and does not appear until the purchase has been effected. Then, if there is a 'case,' he tells them what to say in court, and no wonder 'they often turn out very unreliable witnesses.' It is a shame to employ them for such work at all. The basis of trickery in the whole procedure is highly objectionable, and is unfair to respectable traders. The witnesses employed to give evidence against the latter are most unsatisfactory; while the manner in which their evidence is usually prepared for them is disgraceful." We said the witnesses were "unreliable," and the system "lowering to the dignity of the law," and it seems we were right.

